# Chronic granulomatous disease in the United Arab Emirates: clinical and molecular characteristics in a single center

**DOI:** 10.3389/fimmu.2023.1228161

**Published:** 2023-11-02

**Authors:** Amna Ali Al Kuwaiti, Ahmed Darwaish Al Dhaheri, Moza Al Hassani, Zbigniew Ruszczak, Ahmad Alrustamani, Walid Abuhammour, Gehad El Ghazali, Suleiman Al-Hammadi, Hiba M. Shendi

**Affiliations:** ^1^ Department of Pediatrics, Division of Pediatric Allergy/Immunology, Tawam Hospital, Al Ain, United Arab Emirates; ^2^ Department of Pediatrics, Infectious Disease Division, Sheikh Khalifa Medical City, Abu Dhabi, United Arab Emirates; ^3^ Division of Dermatology, Department of Medicine, Sheikh Khalifa Medical City, Abu Dhabi, United Arab Emirates; ^4^ Department of Medicine, Sheikh Khalifa Medical City, Abu Dhabi, United Arab Emirates; ^5^ College of Medicine, Mohamed Bin Rashid University of Medicine and Health Sciences, Dubai, United Arab Emirates; ^6^ Department of Pediatrics, Al Jalila Children’s Hospital, Dubai, United Arab Emirates; ^7^ Department of Immunology, Sheikh Khalifa Medical City, Union71- Purehealth, Abu Dhabi, United Arab Emirates; ^8^ College of Medicine and Health Sciences, United Arab Emirates University, Al Ain, United Arab Emirates

**Keywords:** chronic granulomatous disease, inborn error of immunity, United Arab Emirates, NCF1, infections

## Abstract

**Background:**

Chronic granulomatous disease (CGD) is a genetic disorder caused by defective oxidative burst within phagocytes, manifesting as recurrent, severe infections as well as hyperinflammation.

**Objective:**

This is the first report from the United Arab Emirates (UAE) to describe the demographic, clinical, laboratory, radiological, and genetic characteristics of patients with CGD.

**Methods:**

This is a retrospective study that was conducted at Tawam Hospital in the UAE on patients with confirmed CGD between 2017 and 2022.

**Results:**

A total of 14 patients were diagnosed with CGD, of whom 13 patients had autosomal recessive (AR) CGD due to NCF1 deficiency. Consanguinity was noted in all patients with AR CGD, whereas positive family history was identified in 50% of cases. The median age of onset of symptoms was 24 months, while the median age at diagnosis was 72 months. Lymphadenitis was the most common clinical feature identified in 71% of patients. Other common infectious manifestations included abscess formation (57%), pneumonia (50%), invasive aspergillosis (21%), oral thrush (14%), and sepsis (14%). Disseminated trichosporonosis was reported in one patient. Autoimmune and inflammatory manifestations included celiac disease in two patients, diabetes mellitus and asymptomatic colitis in one patient each. Genetic analysis was performed in all patients; NCF1 deficiency was diagnosed in 13 (93%) patients, with c.579G>A being the most prevalent pathogenic variant identified. The treatment modalities, as well as treatment of acute infections, treatment modalities included antimicrobial prophylaxis in 12 (86%) patients and hematopoietic stem cell transplant in six patients (42%).

**Conclusion:**

This is the first report from the UAE describing the clinical and molecular characteristics of patients with CGD. The homozygous variant c.579G>A causing NCF1 deficiency can be considered as a founder mutation for AR CGD in the UAE.

## Introduction

Chronic granulomatous disease (CGD) is a rare inborn error of immunity (IEI) caused by defects in the NADPH-oxidase complex, which result in impaired respiratory oxidative species formation within phagocytes, a crucial step in pathogen killing (1). CGD is characterized by recurrent infections, granuloma formation,and other autoimmune and inflammatory manifestations (2, 3). Six known gene defects are associated with CGD. X-linked CGD secondary to *CYBB* (gp91 ^phox^) is the most prevalent and severe form worldwide. The remaining five defects cause autosomal recessive (AR) CGD and include *CYBA* (p22^phox^), *NCF1* (p47^phox^), *NCF2* (p67^phox^), *NCF4* (p40^phox^), and *CYBC1* (1, 4). Among the AR forms of CGD, NCF1 (p47 ^phox^) deficiency is the most prevalent in countries within the Middle East and North African (MENA) region with high rates of consanguinity such as Oman and Saudi Arabia (5, 6). The incidence of CGD within the MENA region is unknown. However, a study from the eastern province of Saudi Arabia reports an estimated incidence of 5.2 per 100,000 (6), exclusively caused by autosomal recessive forms, a 10-fold higher incidence than in the United States (2). A recent review from the MENA registry on IEI reported CGD in 4.8% of cases among their cohort (7).

There is lack of data from the UAE on CGD. To the best of our knowledge, this is the first report on CGD on the UAE population that highlights the burden of the disease and describes the demographic, clinical, laboratory, radiological, and genetic characteristics of patients with CGD. It also discusses similarities and differences between the studied group and other countries within and outside the MENA region.

## Methods

A retrospective chart review was conducted on patients with confirmed CGD who attended the Allergy/Immunology Clinic at Tawam Hospital between 2017 and 2022. During the study period, Tawam Hospital was considered the main referral center for IEI diagnosis and management within the Emirate of Abu Dhabi; patients were also referred from other emirates. In total, 14 patients from nine different families were diagnosed with CGD. A standardized data collection sheet was used to gather demographic, clinical, laboratory, radiological, and genetic data on patients with CGD during the study period. The data were anonymized, and a statistical analysis was performed using Microsoft^®^ Excel ^®^ 2016 MSO (16.0.14026.20276). Continuous variables were presented as median with interquartile range. Categorical variables were presented as frequencies and percentages.

Individual patient consent was not sought as this observational study involved the secondary use of non-identifiable patient information previously collected for routine patient care. Ethical approval was obtained from the Institutional Review Board at Tawam Hospital.

### Dihydrorhodamine test

Dihydrorhodamine (DHR) test was performed using two methods consecutively. Nitroblue tetrazolium test was unavailable. Method 1 involved using the commercial (Phagoburst) test kit (Glycotope Biotechnology GmbH, Heidelberg, Germany). This kit became unavailable in 2021; therefore, method 2, using FagoFlow Ex kit (Exbio Diagnostics, Vestec, Czech Republic) was evaluated and implemented in 2022.

#### Method 1

Heparinized whole blood was collected from patients and incubated with a variety of stimuli including opsonized, unlabeled *E. coli* bacteria, phorbol 12-myristate 13-acetate (PMA), formyl-methionyl-leucyl-phenylalanine (fMLP), and DHR123 (fluorogenic substrate) at 37˚C. A patient sample without stimulus was regarded as negative control, while a sample from a healthy, unrelated control was run simultaneously and served as positive control. A lysing solution was used to lyse the RBCs and partially fix the leukocytes, followed by a washing step using a washing solution. After that, a DNA-staining solution was added to eliminate debris and artifacts. The formation of reactive oxygen metabolites was determined by oxidation of DHR 123 to rhodamine 123. Using FacsDiva flow cytometry (BD biosciences), a live gate was set on granulocytes using forward *versus* side scatter; then, the percentage of the cells that produced reactive oxygen metabolites and their mean fluorescence intensity were calculated (normal reference range was provided as the percentage of oxidized granulocytes by *E. coli*: 95%–100%, fMLP:1%–20%, PMA: 99%–100%). Plots were reviewed. For method 1, the results of the percentage of oxidized granulocytes by PMA are included in **Table 1**. Data on mean fluorescence intensity (MFI) and stimulation index (SI) from method 1 could not be retrieved at the time of data collection.

**Table 1:** Demographics, clinical features, laboratory findings, imaging, and organisms isolated in chronic granulomatous disease population in UAE.

Patient (nationality)	Onset of symptoms (gender)	Clinical presentation	Healthy control DHR (%)/SI	Patient DHR/SI	Imaging	Organism (site cultured)	Gene variant
P1 family 1 (UAE)	14 years (M)	Recurrent abscesses, axillary lymphadenitis, skin ulcers, and fistula, failure to thrive (FTT)	NA	NA	CT chest: atelectatic bands in the right middle lobe and left lingular segment; fistulous inflammatory areas; small pocket of fluid in bilateral supra- and infraclavicular soft tissue	*Staphylococcus aureus* and *E. coli* (skin)	Homozygous pathogenic variant c.579G>A (p.Trp193*) in *NCF1* gene
P2 family 1 (UAE)	2.5 years (F)	Pulmonary TB, lobectomy, recurrent lymphadenitis, FTT, pre-auricular sinusitis, periapical abscess, pustular acne	99.9%^a^	9%^a^	US neck: bilateral cervical lymphadenopathy; infected supraclavicular lymph node CT chest: fibrotic changes in the bilateral apical region, multiple subpleural and centrilobular nodules in the peripheral aspect in the bilateral lungs, S/p right lower lobe lobectomy, pleural thickening in the right lower hemithorax	Negative skin cultures	Homozygous pathogenic variant c.579G>A (p.Trp193*) in *NCF1* gene
P3 family 1 (UAE)	12 years (F)	Hepatic abscess, trichosporonosis, FTT	99.9%^a^	9%^a^	MRI of left leg and pelvis: left iliac bone lytic lesion, consistent with osteomyelitis and osseous destruction, with phlegmon and myositis CT chest: cavitary lung nodule within the left lower lobe with new right-sided lung consolidation US abdomen: heterogenous hypoechoic non-vascular left lobe hepatic focal lesion with internal septation	*Streptococcus intermedius* (liver abscess), *Trichosporon* (bone, BAL)	Homozygous pathogenic variant c.579G>A (p.Trp193*) in *NCF1* gene
P4 family 1 (UAE)	6 months (F)	Recurrent cervical lymphadenitis, recurrent pneumonia, FTT, warts, celiac disease	99.9%^a^	14%^a^	US neck: bilateral cervical lymphadenopathy	*Serratia marcescens* (lymph node)	Homozygous pathogenic variant c.579G>A (p.Trp193*) in *NCF1* gene
P5 family 2 (UAE)	2 years (M)	Recurrent bilateral cervical lymphadenitis, FTT, recurrent pneumonia, septic shock, aphthous ulcers	99.7%^a^	23%^a^	NA	*Chromobacterium violaceum* (blood)	Homozygous pathogenic variant c.579G>A (p.Trp193*) in *NCF1* gene
P6 family 3 (Sudan)	14 months (M)	Recurrent lymphadenitis, scalp and epidural abscesses, recurrent oral thrush, conjunctivitis	100%^a^	4%^a^	MRI brain: thick-walled cystic lesion arising from the left coronal suture with underlying bony defect US neck: multiple-matted lymph nodes in the right side of the neck with loss of normal architecture	*Serratia marcescens* (epidural abscess)	Homozygous pathogenic variant c.678T>G (p.Tyr226*) in *NCF1* gene
P7 family 4 (UAE)	8 years (F)	Perianal abscess, recurrent oral thrush, disseminated aspergillosis (brain, lung, DM, and celiac disease)	NA	13%^a^	NA	*Aspergillus flavus* (brain)	Homozygous pathogenic variant c.579G>A (p.Trp193*) in *NCF1* gene
P8 family 5 (Oman)	12 months (M)	Recurrent cervical lymphadenitis, recurrent pneumonia, invasive pulmonary aspergillosis, recurrent aphthous ulcers, FTT	NA	11%^a^	CT chest: marked volume loss and honeycomb pattern in the parenchyma involving the entire right upper lobe, with pleural thickening, fibrosis, traction bronchiectasis and cavitary lesions within the right upper lobe, cavitatory lesion within the right lower lobe with surrounding ground glass opacities, another ground glass nodule in the left lower lobe, small pulmonary nodules throughout both lungs	*Aspergillus fumigatus*, *Hemophilus influenza* (BAL)	Homozygous pathogenic variant c.579G>A (p.Trp193*) in *NCF1* gene
P9 family 6 (UAE)	6 weeks (M)	Bilateral suppurative otitis media, cellulitis adenitis syndrome, HLH, severe anemia requiring blood transfusion, sepsis	NA	NA	US abdomen: spleen is slightly bulky CT chest: bilateral peribronchial thickening? Bronchitis	Mixed skin flora (ear)	Hemizygous pathogenic variant c.676C>T p.(Arg226*) in *CYBB* gene
P10 family 7 (UAE)	2 years (M)	Recurrent pneumonias, recurrent cervical lymphadenitis with abscess formation, recurrent cellulitis/folliculitis, invasive pulmonary aspergillosis	NA	11%^a^	CT chest: residual scarring and traction atelectasis at the site of the infection. There is residual nodularity and subpleural fibrosis. Bilateral apical fibrosis and pleural thickening. There is bilateral subpleural septal thickening CT neck: There are multiple enlarged lymph nodes with necrosis on the right side neck lymph	NA	Homozygous pathogenic variant c.579G>A (p.Trp193*) in *NCF1* gene
P11 family 8 (UAE)	36 months (F)	Recurrent cervical lymphadenitis, liver abscess, subcorneal pustular dermatosis	SI 23	SI 4.9	CT abdomen and pelvis: large discrete area of low-density change in the right lobe of the liver suggestive of early abscess formation US abdomen: avascular hypoechoic lesions in the right lobe of the liver in keeping with liver abscess	Methicillin-susceptible *Staphylococcus aureus* (liver)	Homozygous pathogenic variant c.579G>A (p.Trp193*) in *NCF1* gene
P12 family 8 (UAE)	Asymptomatic (F)	None	SI 49	SI 10	–	None	Homozygous pathogenic variant c.579G>A (p.Trp193*) in *NCF1* gene
P13 family 9 (UAE)	10 months (M)	Left ear abscess, inguinal abscess, lymphadenitis (submental, submandibular), thigh abscess	NA	NA	US neck: multiple well-defined hypoechoic areas with significant hyperaemia, may represent suppurative lymph nodes US extremity: multiple heterogenous hypoechoic well-defined areas with internal debris and hyperaemic thickened peripheral capsule suggestive of abscesses: one in the right buttock, three in the left buttock	*Serratia marcescens* (skin and ear abscess) *Klebsilla pneumonia* (thigh abscess)	Homozygous pathogenic variant c.579G>A (p.Trp193*) in *NCF1* gene
P14 family 9 (UAE)	Asymptomatic (F)	None	NA	NA	–	None	Homozygous pathogenic variant c.579G>A (p.Trp193*) in *NCF1* gene

NA, not available; US, ultrasound; HLH, hemophagocytic lymphohistiocytosis; DM, diabetes mellitus; FTT, failure to thrive; SI, stimulation index (method 2); DHR, percentage of granulocyte oxidized by phorbol 12-myristate 13-acetate (method 1).

a Method 1.

* Method 1.

- Not required.

#### Method 2

Heparinized whole blood from patients and a healthy, unrelated control were incubated with *E. coli* (as stimulus) and PMA simultaneously, while a patient sample without a stimulus served as negative control. DHR123 was then added to all tubes, and gentle vortexing was applied. The tubes were then incubated for 20 min at 37˚C. Addition of a lysing solution was followed by further incubation for 5 minutes at room temperature. Finally, demineralized water was added to the tubes that were incubated for 5–10 min until the RBCs were lysed. Oxidation of DHR 123 was determined by the production of rhodamine 123 in fluorescein isothiocyanate channel (525 nm) using a flow cytometer. A live gate was set on granulocytes using forward *versus* side scatter on samples stimulated by *E. coli*, PMA, negative control, and healthy control.

The results were reported as SI, defined as the ratio of MFI of granulocytes stimulated by PMA to that of unstimulated granulocytes. A normal cutoff value of SI was considered as >10. Eight patients (P2, P3, P4, P5, P6, P7, P8, and P10) underwent method 1, and their results are reported as the percentage of oxidized granulocytes by PMA (**Table 1**), while two patients (P11 and P12) underwent method 2, and their results are reported as SI (**Table 1**). DHR was not done in the remaining patients, and their diagnosis was confirmed by genetic testing.

### Genetic testing

All patients in our cohort underwent genetic testing in the form of whole-exome sequencing or targeted gene sequencing when a known variant was previously identified in a sibling/family member. Samples were sent to out source laboratory.

## Results

### Patient characteristics

A total of 14 patients with confirmed CGD (**Table 1**) based on clinical features, DHR assay, and/or detection of pathogenic variants in genes encoding NADPH–oxidase complex were enrolled in the study. DHR was performed in 10 out of 14 patients. AR CGD was diagnosed in 13 (93%) patients from nine different kindreds, while X linked-CGD (X-CGD) was identified in one patient. The cohort included seven male patients and seven female patients. Moreover, 86% (*n* = 12) of our cohort were Emirati nationals, while 14% (*n* = 2) were non-Emirati (Oman, *n* = 1 and Sudan, *n* = 1). All patients with AR CGD (*n* = 13, 93%) were born to consanguineous parents, whereas positive family history was reported in seven patients (50%). The median age of onset of symptoms was 24 months (IQR: 10–36 months), whereas the median age at diagnosis was 72 months (IQR: 14.1–150 months). The median diagnostic delay was 36 months (IQR: 3–64.5 months) with a median follow-up duration of 13.8 months.

### Clinical profile

**Tables 1**, **2** delineate the clinical, laboratory, and radiological findings of patients with CGD. The most prevalent clinical manifestation was lymphadenitis (*n* = 10, 71%), mainly cervical (*n* = 8), axillary (*n* = 1), cellulitis adenitis syndrome (*n* = 1) followed by abscess formation (*n* = 8, 57%), mainly skin (*n* = 4, liver *n* = 2, epidural *n* = 1, and perianal *n* = 1). Pneumonia (*n* = 7, 50%) and failure to thrive were the next common features. Invasive aspergillosis was encountered in three cases, two of whom developed invasive bronchopulmonary aspergillosis (**Figure 1**), and one had disseminated aspergillosis with intracerebral abscess formation. Oral thrush and sepsis occurred in equal frequencies (14%). Disseminated trichosporonosis with osteomyelitis and pulmonary tuberculosis (TB) occurred in one patient each (P3 and P2, respectively).

**Table 2:** Clinical features of the chronic granulomatous disease population in UAE.

	*N* (%)
Infectious manifestations	
Lymphadenitis	10 (71)
Abscess	8 (57)
Pneumonia	7 (50)
Invasive aspergillosis -Pulmonary -Disseminated (pulmonary aspergilloma and brain abscess)	3 (20) 2 (14) 1 (7)
Oral thrush	2 (14)
Sepsis	2 (14)
Disseminated trichosporonosis	1 (7)
Pulmonary TB	1 (7)
Suppurative otitis media	1 (7)
Warts	1 (7)
Conjunctivitis	1 (7)
Non-infectious manifestations	
Failure to thrive	6 (42)
Celiac disease	2 (14)
Aphthous ulcers	2 (14)
Diabetes mellitus	1 (7)
Colitis	1 (7)
Fulminant hemophagocytic lymphohistiocytosis	1 (7)
Anemia	1 (7)
Skin ulcer and fistula	1 (7)

**Figure 1:**
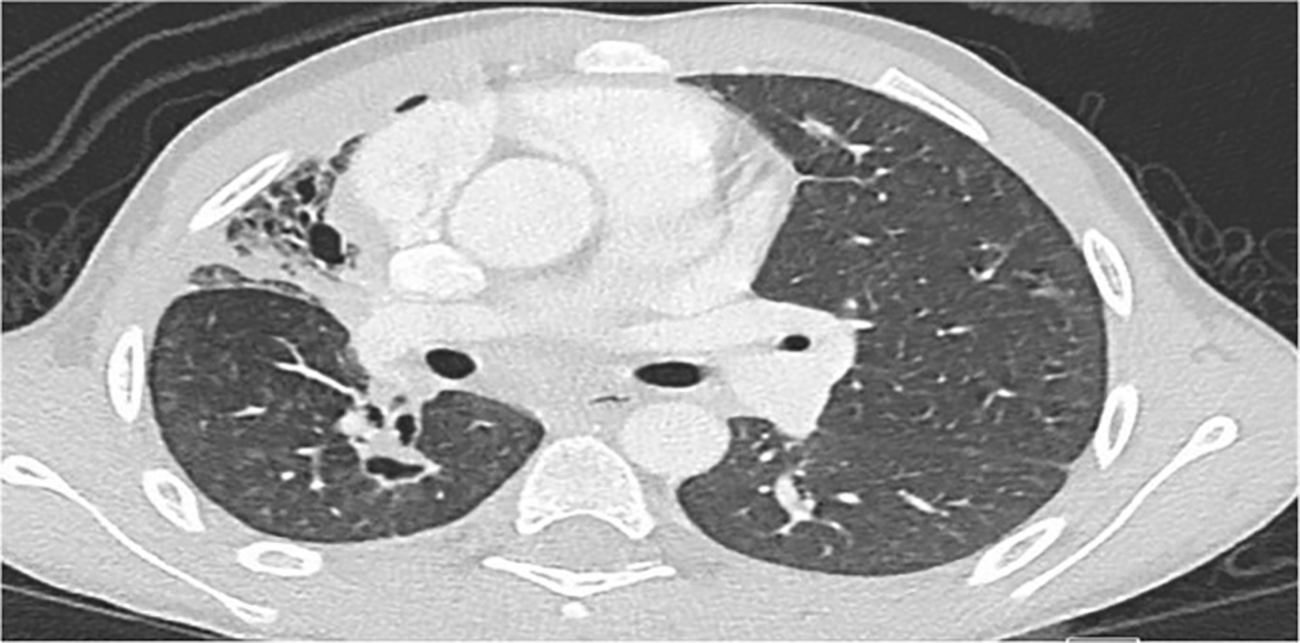
Chest CT in P8 shows marked volume loss and honeycomb pattern in the parenchyma involving almost the entire right upper lobe, associated with pleural thickening. Linear densities in the right lower lobe and left upper lobe suggestive of fibrotic changes. Small cavitating lesion in the apex of the right lower lobe is noted.

**Figure 2:**
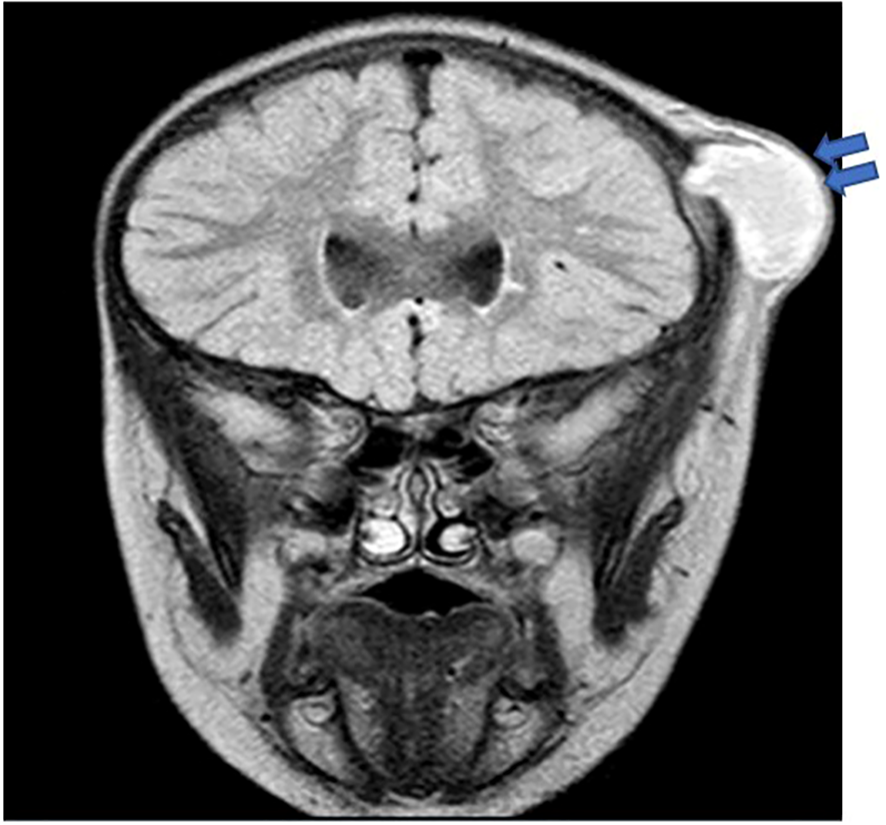
Brain MRI in P6 revealed a thick-walled cystic lesion (arrows) with some layering of signal arising from the left coronal suture with an underlying bony defect. No intracranial extension, although there is pachymeningeal enhancement underneath the lesion.

In terms of inflammatory/autoimmune manifestations, P4 and P7 were diagnosed with celiac disease, P7 also had diabetes mellitus with negative anti-islet cell and anti-GAD antibodies, while P5 was diagnosed with asymptomatic colitis by endoscopy during pretransplant evaluation. Sadly, P9, with X-linked CGD, developed fulminant hemophagocytic lymphohistiocytosis (HLH) at 6 months of age. Two patients (P12 and P14) were asymptomatic at diagnosis. They were evaluated by genetic testing early in life as both had an affected sibling. With regards to infective microorganisms, *Serratia marcescens* was the predominant bacterial pathogen isolated from an epidural abscess (**Figure 2**), lymph node, and skin abscesses from three different patients (P6, P4, and P13, respectively). *S. aureus* and *Escherichia coli* were detected from the skin of P1, while *S. aureus* was recovered from a liver abscess in P11. *Streptococcus intermedius* was identified as the causative pathogen for liver abscess in P3, while *Chromobacterium violaceum* was isolated from blood culture in P5. *Aspergillus fumigatus* and *Haemophilus influenzae* were detected from broncho-alveolar lavage (BAL) in P8, whereas *Aspergillus flavus* was isolated from brain tissue in P7. Another fungal pathogen, *Trichosporon* sp., was isolated from BAL, lung, and bone biopsy in P3 (**Table 3**).

**Table 3:** Type of microorganisms isolated in chronic granulomatous disease population from UAE.

Organism	Site	*N* (%)
*Serratia marcescens*	Epidural, lymph node, skin	3 (21)
*Staphylococcus aureus*	Skin, liver	2 (14)
*Escherichia coli*	Skin	2 (14)
*Chromobacterium violaceum*	Blood	1 (7)
*Streptococcus intermedius*	Liver	1 (7)
*Klebsiella pneumoniae*	Skin	1 (7)
*Haemophilus influenzae*	BAL	1 (7)
*Aspergillus fumigatus*	BAL	1 (7)
*Aspergillus flavus*	Brain	1 (7)
*Trichosporon* sp.	Bone, BAL	1 (7)

### Immunological profile

**Figure 3:**
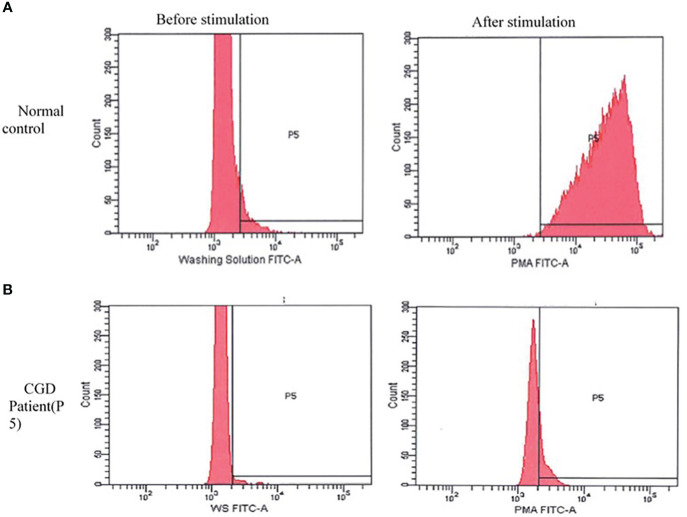
Dihydrorhodamine pattern before and after stimulation with phorbol 12-myristate 13-acetate (PMA). **(A)** Normal pattern in healthy control: shows a shift in the curve to the right after stimulation with PMA. **(B)** The curve of the chronic granulomatous disease patient (P5) remain unchanged after stimulation. A similar pattern was seen in other patients.

DHR was significantly impaired in 10 patients in whom the test was undertaken when compared to healthy controls (**Figure 3**).

Serum immunoglobulin levels and lymphocyte subsets were checked at presentation on most patients to exclude other IEIs. Most patients had unremarkable results, except one patient who had mild T cell lymphopenia.

### Molecular profile

The same homozygous variant c.579G>A in *NCF1* gene was identified in 12 out of 13 patients with AR CGD. One patient had a different homozygous variant c.678T>G in *NCF1* gene. The diagnosis of X-CGD was established in one patient with a hemizygous variant c.676C>T in *CYBB* gene.

### Management and outcomes

Prophylactic trimethoprim–sulfamethoxazole and itraconazole were commenced in 11 out of 14 patients. One patient, P3, also received interferon gamma as adjunctive therapy after developing trichosporonosis.

Six patients (42%) underwent hematopoietic stem cell transplantation (HSCT) outside UAE, and one passed away due to HSCT complications (P1). The patient with X-CGD (P9) passed away secondary to fulminant HLH at 6 months of age before the diagnosis of CGD was established. The results of the genetic testing were obtained following his death.

## Discussion

This study is the first study to describe the phenotype and genotype of CGD in the UAE. In a recent study published on the spectrum of IEI in the UAE, congenital defects of phagocyte number or function accounted for 8.4% of IEI, with CGD being the most prevalent disorder (8). **Table 4** demonstrates the similarities and difference between different populations with CGD across the world. Most of our patients (93%) had AR CGD due to NCF1 deficiency. This is explained by the high level of consanguinity in UAE like other countries within the MENA region, such as Oman (5), Saudi Arabia (6), Turkey (12), Iran (13) and Egypt (15). Although X-linked CGD is the most common form of the disease in the United kingdom, Latin America, Mexico, and USA (2, 10, 11, 16), it was only observed in one patient in our cohort.

**Table 4:** Demographics, clinical features, laboratory findings, organisms isolated, and genetic results among chronic granulomatous disease population (CGD) patients across the world.

Population	USA	Europe	Latin America and Mexico	UAE	Turkey	Iran	Saudi Arabia	India
Age of onset in months (mean/median)	NA	NA	23.9 3 in Mexico	47.1	12	20^a^ (AR CGD) 5 (X-CGD)	42	8^a^
Age at diagnosis in months (mean/median)	36 (X-CGD) 93.6 (AR)	58.8(X-CGD) 105.6 (AR)	52.7 30 in Mexico	95.6	32.4 (X-CGD) 62.4 (AR)	69.6(AR CGD) 11(X-cGD)	54	24
Common clinical feature	Pneumonia	Pneumonia	Pneumonia	Lymphadenitis	Pneumonia	Lympadenopathy	Lymphadenitis	Pneumonia
Isolated organism (site)	*Aspergillus* spp. (lung, brain), *S. aureus* (skin, liver, lymph node), *Serratia* spp. (bone)	*Staph aureus*	*S. aureus*	*Serratia* spp.	*Aspergillus* spp. (lung), *S. auerus* (lymph node, skin, liver, and lung)	*Aspergillus* spp. (lung, brain)	*S. aureus*	*S. aureus*
Inflammatory/autoimmune manifestation	Colitis (17.4%), obstructive lesion GI and GU (26.4%), lupus, ITP, choretinitis, myasthenia gravis	Obstructive lesion GI and GU (10%), colitis (9%), lupus, RA, dermatomyositis, sarcoidosis, ITP, autoimmune hepatitis	Colitis (19.4%)	Celiac disease (14.3%), DM, colitis, HLH	Stomatitis (43%), colitis (4.5), reactive arthritis, autoimmune hepatitis, ITP, and pericardial effusion	Lupus (5.4%), RA (3.23%), autoimmune enteropathy, chorornitis, and ITP	Colitis (18.2%)	Lung granuloma (6.8%), colitis (5.1%), liver granuloma, HLH, HLA-B27-related arthritis, Kawasaki disease, intestinal obstruction
Mycobacterial disease	Mycobacterial disease (4%) and atypical mycobacterial (2%)	Adverse reaction to BCG (8%)	Adverse reaction to BCG (30%) in Latin America. In Mexico adverse reaction to BCG (56%) and mycobacterial disease (29%)	1 treated for pulmonary TB	Adverse reaction to BCG (22.5%)	Mycobacterial disease (16%), adverse reaction to BCG (55.9%)	Adverse reaction to BCG (9.1%)	Mycobacterial disease (18.6%), adverse reaction to BCG (38.6%)
Genotype	CYBB (70%), AR (22%), unknown (8%)	CYBB (67%), AR (33%)	CYBB (74.6%), AR (25.5%) in Latin America; CYBB (77%) and AR (33%) in Mexico	CYBB (7%), AR (93%)	CYBB (38%), AR (62%)	CYBB (12.9%), AR (87.1%)	AR (100%)	CYBB (44.7%), AR (55.6%)
Common AR form (variant detected)	NCF1 deficiency (56%) (c.75_76 del.GT)	NCF1 deficiency (49%) (NA)	NCF1 deficiency (22.5%) (c.75_76 del.GT) Latin America; NCF1 and NCF2 in Mexico (8.5%)	NCF1 deficiency (93%) (c.579G>A)	CYBA deficiency (22.5%) (c.166dupC)	NCF1 deficiency (55%) (NA)	NCF1 deficiency (100%) (c.75_76delGT)	NCF1 deficiency (31.9%) (c.75_76delGT)
Mortality	17.6%	20%	39.4% (Mexico)	14%	10%	9.7%	NA	38.5%
Reference	Winkelstein et al. (2)	van den Berg JM et al. (9)	de Oliveira-Junior et al. (10), Blancas-Galicia et al. (11)	Our study	Köker et al. (12)	Fattahi et al. (13)	Suliaman et al. (6)	Rawat et al. (14)

NA, not available; ITP, immune thrombocytopenic purpura; SLE, systemic lupus erythematosus; GI, gastrointestinal; GU, genitourinary; RA, rheumatoid arthritis; HLH, hemophagocytic lymphohistiocytosis.

a Median.

The median age at diagnosis in our cohort was 6 years, younger than that reported in Iranian patients with NCF1 deficiency where the median age was 10 years (17). However, the median age at diagnosis was only 24 months in an Indian cohort that collectively included AR and XL forms of CGD (14). X-CGD is known to present at an earlier age with severe manifestations (2). Unfortunately, our patient with X-linked CGD developed fatal HLH early in life.

Lymphadenitis was the most common presenting clinical feature among our group (71% of cases) like Saudi Arabia (6), whereas abscess formation was the predominant clinical feature in the case series from Iran (17) and Malaysia (18). This is in contrast to USA (2), Europe (9), Latin America (10), Mexico (11), Turkey (12), and India (14), as pneumonia was the most observed presenting clinical manifestation. Although *Staphylococcus aureus* was the most prevalent bacteria identified in patients with CGD in other series (9–11, 14, 17–19), our microbiological profile revealed that *Serratia marcescens* was the main isolated bacterial pathogen.

Comparable with other studies, *Aspergillus* species was the prevalent fungal microorganism detected in our cohort (16, 18, 20, 21).

In the UAE, the *Bacillus* Calmette–Guerin (BCG) vaccine is administered at birth as part of the national vaccination program. Although BCG-related complications are not uncommon in CGD (12, 14, 17), none of our patients had a confirmed adverse reaction to BCG. One patient in our cohort was diagnosed with pulmonary tuberculosis, requiring lobectomy at the age of 2.5 years. The culture results could not be obtained; it was therefore not possible to determine if the infection was due to BCG or *Mycobacterium tuberculosis*. In other countries that undertake the same practice such as Latin America, Mexico (10, 11), and India (14), the complications due to BCG in CGD patients were 30%, 56% and 38.6%, respectively.

Pyogenic liver abscess formation secondary to *Streptococcus intermedius* is considered rare, with only a few cases reported in patients with CGD (22–25). Both *Streptococcus intermedius* and methicillin-susceptible *Staphylococcus aureus* emerged as causative organisms for liver abscess in one patient each in our cohort. A rare aggressive fungal organism that was detected in our cohort was *Trichosporon* species, previously reported in six patients with CGD, four of whom presented with pneumonia (26–28), while one patient developed lung abscesses (29) and one patient was diagnosed with multiple brain abscesses (30). Most patients required surgical resection despite antifungal therapy. However, in the patient with brain abscesses, complete surgical resection was unfeasible due to the critical site of the lesions, so the patient was managed with dual antifungal therapy that resulted in complete resolution and recovery. P3 experienced prolonged cough with left hip and leg pain. Investigations revealed a high ESR level (108 mm/h), and the CT chest demonstrated cavitation involving both the upper and lower segments of the lower left lung lobe. Additionally, MRI of the left leg and pelvis revealed lytic lesions of the left iliac bone, phlegmon, and myositis—findings that were consistent with osteomyelitis and osseous destruction. Cultures from BAL, lung biopsy, and bone biopsies grew *Trichosporon* species. The patient was managed with dual antifungal therapy (posaconazole and lipososomal amphotericin B) as well as adjunctive interferon gamma therapy prior to HSCT.

The routine use of interferon gamma prophylaxis remains controversial. It is a more widely implemented practice in the USA compared to Europe (31). A multicenter study on the use of interferon gamma in CGD patients demonstrated 77% reduction in serious infections compared to the placebo group: the effect was more pronounced in the younger age group when combined with antimicrobial prophylaxis (32). Interferon gamma is not routinely used in UAE, as it is not readily available and is costly.

Among the autoimmune and auto-inflammatory manifestations noted in our group were celiac disease, colitis, diabetes mellitus, and secondary HLH. Compared with other cohorts with a predominance of AR CGD as in Turkey (12), Iran (13), and India (14), asymptomatic colitis was diagnosed in one patient and was treated successfully with budesonide and rifaximin.

Celiac disease was diagnosed in two patients in our cohort before the diagnosis of CGD was established. One patient had a high level of IgA anti-tissue transglutaminase antibodies and evidence of villous atrophy with intraepithelial lymphocytosis on duodenal biopsy. The second patient was diagnosed with both celiac disease and insulin-dependent diabetes mellitus at another emirate prior to the CGD diagnosis. The electronic records of the second patient were unavailable to us. We could not locate any previous reports on the association of celiac disease with CGD.

A retrospective study done at the NIH (33) on patients with CGD reported the incidence of diabetes as 9.4%, primarily in NCF1 deficiency regardless of the mutation or residual superoxide activity. One patient in that cohort was tested for anti-islet, insulin, and GAD antibodies and had negative results, similar to our patient.

In a retrospective study on HLH in IEI, HLH was noted in around 35% of patients with CGD (34), predominantly in X-CGD. P9, who was later diagnosed with X-CGD had clinical and laboratory features (persistent fever, splenomegaly, pancytopenia, hyperferritinemia, and a high level of CD25) consistent with HLH that was refractory to therapy with antibiotics, intravenous immune globulin, corticosteroids, and anakinra.

Molecular analysis was performed in all patients. The results showed that 93% of our patients had NCF1deficiency. The homozygous, pathogenic variant c.579G>A (p.Trp193*) in *NCF1* was detected in 12 out of 13 patients with AR-CGD; 11 were Emirati, and one was from Oman. A different homozygous, pathogenic variant c.678T>G (p.Tyr226*) was identified in P6 who was from Sudan.

The c.579G>A variant in *NCF1* was also reported as the most common variant in neighboring Oman (5). However, c.75_76delGT (35, 36) was the predominant variant associated with NCF1 deficiency in other countries within the MENA region such as Saudi Arabia and Iran as well as further afield in Latin America, India, and Europe (6, 9, 11, 14, 17). A hemizygous pathogenic variant c.676C>T p.(Arg226*) in *CYBB* was detected in P9 with X-CGD.

Prophylactic antimicrobial therapy with trimethoprim–sulfamethoxazole and itraconazole was initiated in all patients except two; one traveled abroad, while the other passed away before the CGD diagnosis was made.

HSCT for CGD is increasingly considered as the curative therapeutic option with good overall survival and lower rejection rates (37). Six of our patients (42%) underwent HSCT abroad, as it was unavailable in the UAE at that time. Out of those, one passed away, two are being followed up in other centers, and two are doing well, while one developed thrombocytopenia post-HSCT, which was managed with steroids and cyclosporine. The remaining patients have been evaluated for HSCT, of whom three are currently abroad for evaluation. No suitable donor was identified for the rest. The overall mortality in our cohort was 14%, which was comparable to a European cohort (9) but lower than that reported in India (30%) (14), Malaysia (25%) (18), and China (43%) (38).

This study does not reflect the exact prevalence of CGD in the UAE which is likely to be under-represented, as this is a single-center study. The milder phenotype of AR CGD and lack of awareness among health professionals about CGD likely limit the referral of these patients to clinical immunologists.

## Conclusion

This is the first study from the UAE to describe the clinical, laboratory, and molecular characteristics of patients with CGD. Autosomal recessive CGD due to NCF1 deficiency is the most prevalent form in the UAE. Our findings are comparable to other countries from the MENA region with a high prevalence of AR CGD and a predominance of infectious manifestations. Multicenter studies and the establishment of a national registry will help in determining the exact burden of the disease.

## Data availability statement

The original contributions presented in the study are included in the article/supplementary materials. Further inquiries can be directed to the corresponding author.

## Ethics statement

This study was reviewed and approved by Institutional Review Board (IRB) at Tawam Hospital. The study was conducted in accordance with the local legislation and institutional requirements. Written informed consent for participation was not required from the participants or the participants’ legal guardians/next of kin in accordance with the national legislation and institutional requirements.

## Author contributions

AK: study design, data collection and analysis, writing and reviewing of the manuscript. HS: patient diagnosis and management, study design, and responsibility for the final manuscript. AD: data collection and review of the manuscript. MH, ZR, and AA: patient management and review of the manuscript. GG, SH, WA: patient diagnosis and review of the manuscript.
